# COVID-19: average time from infection to death in Poland, USA, India and Germany

**DOI:** 10.1007/s11135-022-01340-w

**Published:** 2022-02-15

**Authors:** Antoni Wiliński, Łukasz Kupracz, Aneta Senejko, Grzegorz Chrząstek

**Affiliations:** 1 WSB University, Gdansk, Poland; 2 Koszalin University of Technology, Koszalin, Poland

**Keywords:** Covid-19, Time series, Confirmed infection cases, Correlation, Bootstrapping

## Abstract

There are many discussions in the media about an interval (delay) from the time of the infections to deaths. Apart from the curiosity of the researchers, defining this time interval may, under certain circumstances, be of great organizational and economic importance. The study considers an attempt to determine this difference through the correlations of shifted time series and a specific bootstrapping that allows finding the distance between local maxima on the series under consideration. We consider data from Poland, the USA, India and Germany. The median of the difference’s distribution is quite consistent for such diverse countries. The main conclusion of our research is that the searched interval has rather a multimodal form than unambiguously determined.

## Introduction

The work is written in January 2021. This is quite important information due to the dynamically changing situation of a pandemic from the statistics perspective. The authors do not have data on the distribution of time intervals between the confirmed SARS-CoV-2 infection and death of a given patient. These cases relate to a relatively small group of infected people and are published as a Case Fatality Rate (CFR) indicator, for example on the CSSE JHU website [https://gisanddata.maps.arcgis.com/apps/opsdashboard/] or in “Worldometer—real time world statistics” [worldometers.info].

**Fig. 1:**
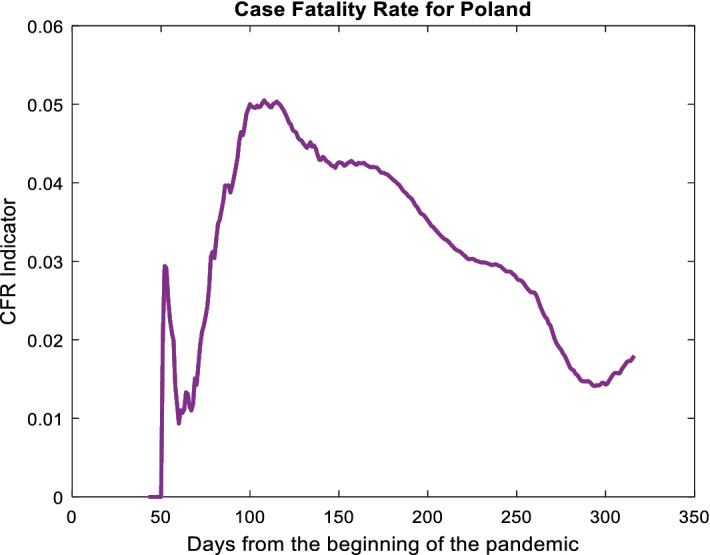
CFR indicator for Poland

This indicator varies quite significantly depending on many factors, some of them are quite obvious like the state of health care, the average age of the society and on the other hand, many of them are hidden. The highest relative number of deaths is recorded today in Yemen (29%, January 2021), whereas in European countries in Lombardy, Italy, it is exceeding 5%, for Poland, Germany and France it is approximately 2–2.5%, in case of Scandinavia it is about 1%, in India from 0.8 to 3%, in the USA from 0.7 to 3.9%, but in the vast majority of states it is about 1%. Despite these small relative indicator’s values, the rate is clearly fluctuating and local increases in the number of deaths may contribute to organizational bottlenecks in the healthcare system due to low system efficiency. For example, independent media has reported in Poland (2–3 months ago–autumn 2020) queues of ambulances transporting patients waiting in front of emergency arrivals or sent from hospital to hospital. If we consider the information that while the relatively small CFR applies to the entire society, the conditional CFR in the group of the most at-risk elderly people may be much greater. Initially, CFR for Poland was quite high and exceeding 5% (during the spring 2020), currently it is relatively low (Fig. 1). This is probably the effect of increased social responsibility, awareness, isolation and security. Another aspect of the relation between the number of infected and the number of deaths is their relation over time. The assessment of the predicted average number of deaths and the time after which this average will occur is interesting for many reasons. Society’s level of morale is also important, because it is significantly impacted by the organizational efficiency and credibility of the state (Scortichini, M. et al. 2020).

There are thousands of publications on the statistical aspects of COVID-19 so far, and we can find the studies directly related to the topic of the article. Chinese medical doctors were interested in these problems at the very beginning of the pandemic in Wuhan. Initially, they used a small group of several hundred infected and consequently dead people (Zhou F. et al. 2020). Interesting statistical research on the issue of lagged infections and deaths over time was also carried out by James et al. (2020) and James & Menzies (2020). The current issue is taken up by Maleki, Mahmoudi, Heydari, & Pho, (2020aandb). It seems that the synthesis of these two articles would allow drawing similar conclusions. Confirmed cases and deaths time series modelling in relation to India are dealt with by Salgotr et al. (2020) and also Elmousalami and Hassanien, (2020) and Zeroual et al. (2020). A similar model is also used by Nakamura et al. (2020) by examining cumulative death curves. The models we consider here are closer to the regressive considerations, which can be seen in the works of Ballı (2020) or Jones (2021). Chrusciel and Szybka (2020) also Chrusciel and Szybka (2021) discuss a very similar topic of the time shift between infections and deaths. They see this issue as difficult to unambiguously solve and giving quite divergent results for different countries. Generally, they believe that the task is impossible to solve in most cases due to the wide variation in the results. Another important approach are models based on the SIR scheme and its gaining accuracy derivatives, such as in the work of Munoz-Fernandez et al. (2021). Often, the issues of confirmed case curves and the number of deaths appear when solving the predictions of virus spread. For example, Oliveira and Moral (2020) make forecasts (short-term) based on data from countries grouped for predictive purposes. A slightly different approach was proposed by Medeiros et al. (2020) using data from countries where the virus appeared earlier in forecasts for Brazil.

There are many valuable works that deal with the social and economic consequences of the pandemic. Sotis (2021) and Venetoklis (2021) write about the dramatic consequences for the entire world in the form of a decline in GDP, an increase in unemployment, poverty and exclusion. Pileggi et al. (2021) also lists such factors having a global impact as the impact on health, the environment and human rights. Uzunar G. (2020) writes about the disastrous impact on tourism with the example of Italy. There are thousands of such sources. In order to emphasise the global significance of the phenomenon, we list several aspects (Jiang et al. 2020), that are maybe not the most important ones.

The article is organized in the following way: we describe a method allowing to determine an interval between infections and deaths. A strong influence of 7-days period is emphasized due to the social circumstances, but not the statistical ones. Then, we describe bootstrapping technique allowing to achieve the final goal of this work. ‘Results’ section contains intervals’ histograms of all 4 considered countries. The work is closed by ‘Discussion’ and ‘Conclusions’ sections.

## Description of the searching method for a time interval between infections and deaths

Looking for a statistical method that would allow to establish the relationship between the number of infected and the number of deaths, the first idea is to check the correlation between these two time series—hereinafter referred to as Conf (from confirmed cases) and Dea (deaths). A fairly obvious method will be to look for the maximum positive correlation on shifted series.

**Fig. 2:**
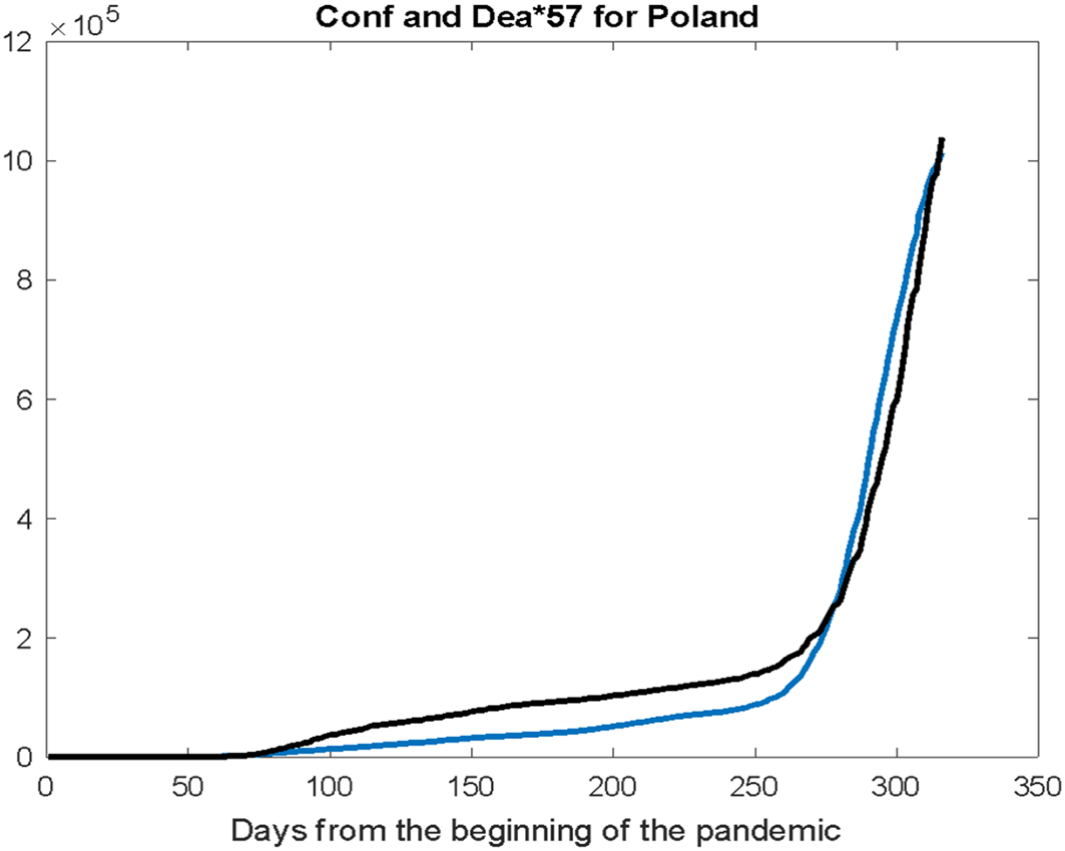
The probability of cumulative values runs: Conf (blue) and Dea increased k = 57 times (black)

While looking for these cumulative cases curve offset of Conf (Confirmed Cases) and Dea (Deaths), we will not find these differences. In Fig. 2 these two curves have been Case Fatality Rate overlaid in order to catch possible patterns. The ordinates of the Dea curve were successively multiplied so as to get as close as possible to Conf. The empirically selected multiplier turned out to be the number k = 57 allowing for the visualization of their similarity. From the programming point of view, we use a simple loop to analyse the sum of the absolute values of several ordinates—a distance between the curves—and to minimize it.

There were no hints obtained according to the offset of the two time series Conf and Dea from the depicted runs. The only measurable result of comparing the Conf and Dea curves in Fig. 2 is the empirical determination of the k coefficient, which will be used later in this work.

**Fig. 3:**
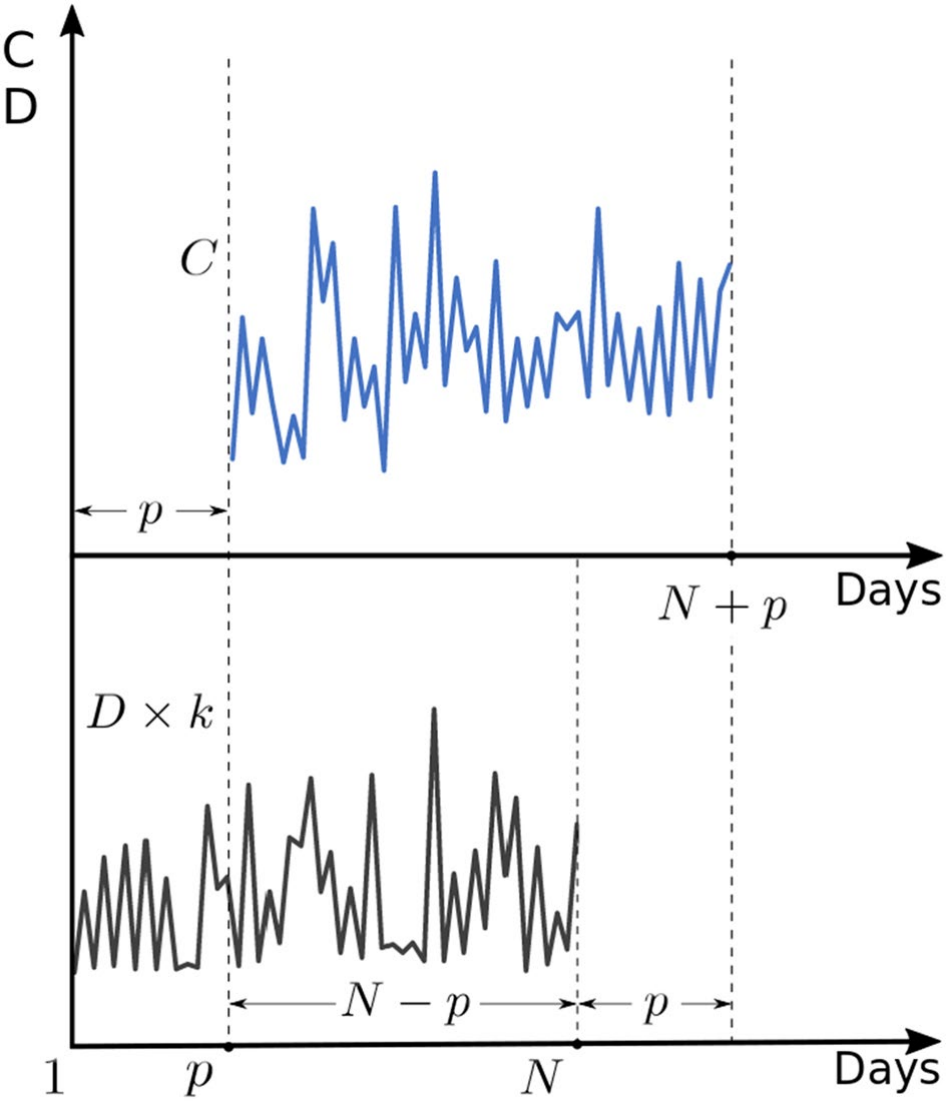
An example of two considered time series Conf and Dea (Dea with symbolically k-times increased values of ordinates) with a shift of the Dea series to the left

Much more information is obtained on the Conf and Dea charts, but for daily increments. Figure 3 shows two time series runs shifted by 7 days. The value of this shift minimizes the sum of the absolute differences between these curves.

Let ${C}_{i}, i=\mathrm{1,2},\dots ,N$ denote Conf time series with $N$ terms and ${D}_{i}, i=\mathrm{1,2},\dots ,N$ Dea time series with $N$ terms, where $i$ is the index of the measurement day (date). There are many publications on COVID-19 using time measurement in days counted from the date of registration by the CSSE [https://gisanddata.maps.arcgis.com/apps/opsdashboard/], i.e. from January 22, 2020. In this situation, $N$ may be the current date.

Now let’s shift the $$D$$ series $$p$$ days backward. We multiply the ordinates of the series $$D$$ by the aforementioned multiplier $$k = 57$$. Then the element $${D}_{1}$$ appears at the $$p + 1$$ position of the series, the element $${D}_{2}$$ at the $$p + 2$$ position, etc. At the $$N$$ position there will be the $$N-p$$ element. If we shift the series $$D (as mentioned above)$$ by $$p = 7$$ days and compare it with the non-shifted series $$C$$, we get the possibility of comparing the local changes of these series presented in Fig. 3.

The computational goal is to find the values of the $p$ and $k$ parameters that minimize the distance between the shifted curves:

1 $$\left( {p,k} \right) = \arg \min \mathop \sum \limits_{i = 1}^{i = N - p} \left| {C_{i + p} - D_{i} .k} \right|$$

This approach will prove the best fit of the both curves, precisely—their (N-p) wide fragments. Perhaps it will allow to determine studied offset of deaths curve in relation to infections curve. During the best fit of the curves, $k$ values were incremented by 1 between values 1 and 100, $p$ values were incremented by 1 in the range from 1 to 15 by 1 day.

The minimum of the distance was found for the parameters $p = 7$ and $k = 57$ for the entire available fragment of series from $i = 1$ to $N-p$ for the series ${D}_{i}$ and from $i = p$ to $N$ for the series $C$.

**Fig. 4:**
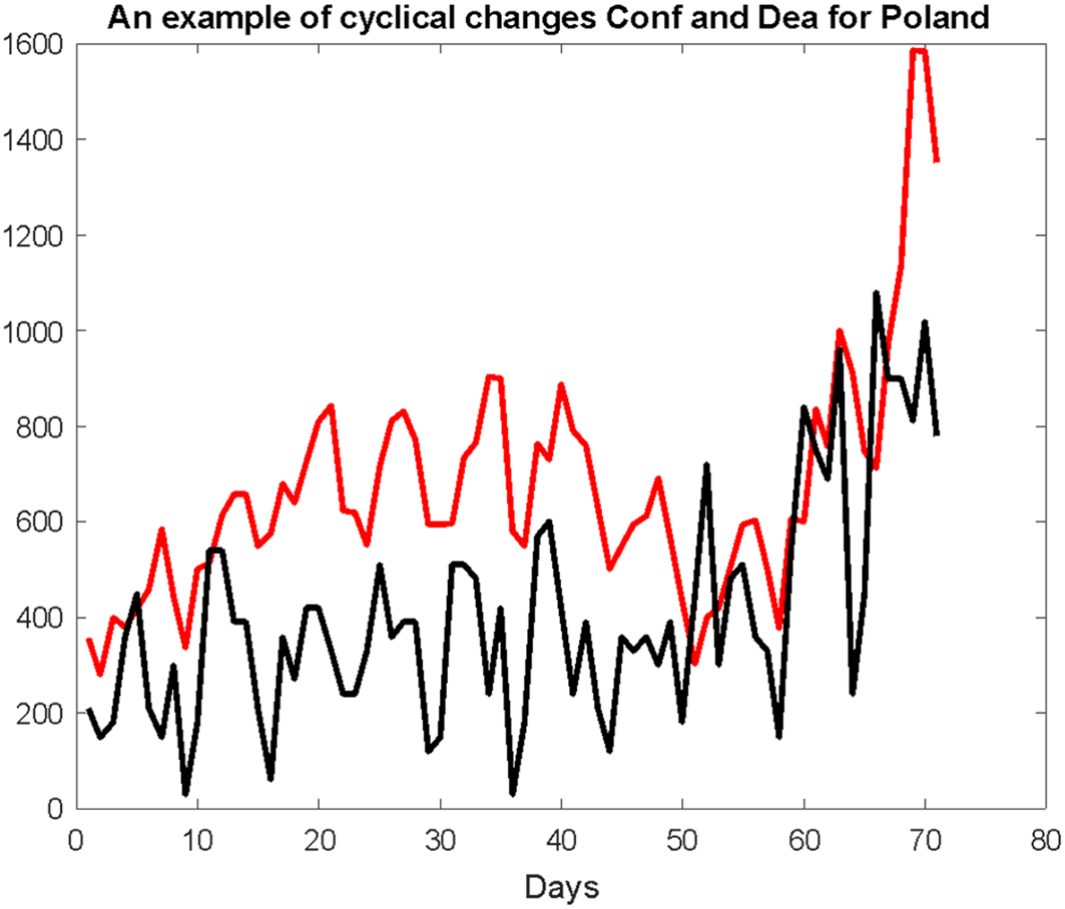
Daily increments of the Conf variable (red curve) and the Dea variable (black curve) with the ordinates increased $\mathrm{k}$ times. The Dea curve is shifted 7 days to the left

The $p = 7$ shift value is quite a characteristic quantity, it is simply a week-time that causes a natural cyclicality of changes in the runs of both these series around the world (Fig. 4). In this context, a difference of the curves’ offset equal exactly to 7 is at least suspicious to dependent on the natural weekly life rhythm of the almost all societies around the world.

The weekly period, mostly often Saturday and Sunday, leads to decrease of the registered infection and death cases in almost all of the cultures. This is most often due to the reduced work intensity of all institutions operating in the health service.

For such shifted time series, their linear Pearson correlation was checked. The correlation coefficient for the shifted series was calculated according to the formula (2):

2 $$r_{{{\text{CD}}}} = \frac{{\mathop \sum \nolimits_{i = 1}^{i = N - p} \left( {C_{i + p} - \overline{C}} \right) \cdot \left( {D_{i} - \overline{D}} \right)}}{{\sqrt {\mathop \sum \nolimits_{i = 1}^{i = N - p} \left( {C_{{{\text{irp}}}} - \overline{C}} \right)^{2} } \cdot \sqrt {\mathop \sum \nolimits_{i = 1}^{N} \left( {D_{i} - \overline{D}} \right)^{2} } }}$$

and here, the average value of daily increases in infections on the Conf curve:

$$\overline{C} = \frac{1}{N - p}\mathop \sum \limits_{i = p}^{i = N} C_{i}$$

and, the average value of the daily deaths:

$$\overline{D} = \frac{1}{N - p}\mathop \sum \limits_{i = 1}^{i = N - p} D_{i}$$

**Fig. 5:**
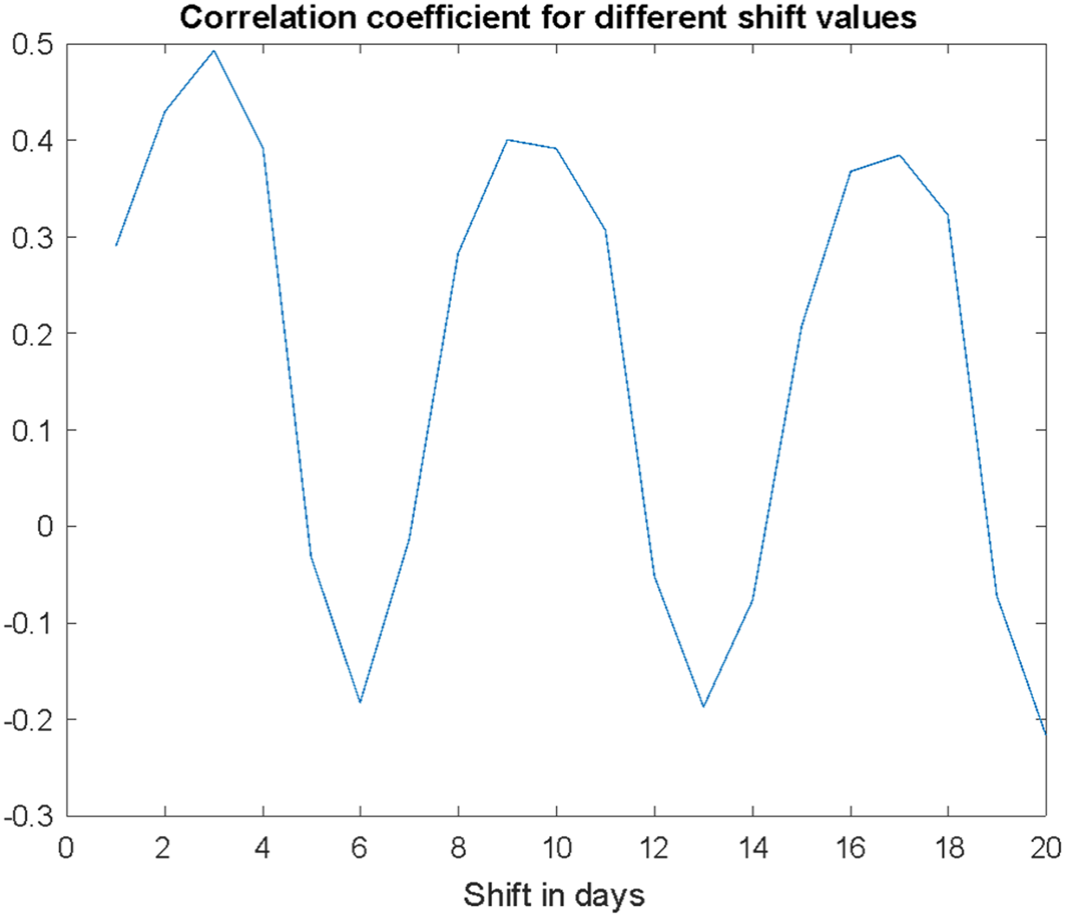
The correlation coefficient for the two considered series in days Conf and Dea for different values of the variable shift—series Dea forwards to Conf [in days]—data for Poland

The changes of the correlation coefficient as a function of the shift are presented in Fig. 5. Regardless of the value of the correlation coefficient (the longer the time window, the higher), the chart clearly shows cycles with a period of 7 days (on the data for Poland).

These changes most often amount to a significant reduction in both the number of confirmed cases and registered deaths. This most often happens due to the reduced work intensity of all institutions operating in the health service.

Chart 5 shows a clear cyclicality of the correlation coefficient depending on the size of the shift. The first maximum occurs at a random place (here for $i = 3$) depending on where the correlation calculation is started. However, the next ones have a regular cycle with a period equal to 7. This is another confirmation of the strength of the weekly influence on the interdependence between Conf and Dea. These weekly maximums and minimums do not reveal the analysed relationship between the local maximums on the Conf curve and their consequences on the Dea curve. What can be seen in Fig. 5 is rather a correlation caused by the minimums of testing and infection registration falling out at weekends due to lower activity of the institutions. Thus, what is the real relationship and the real shift between the maxima?

There will be different approaches presented.

Rather, the question should be posed as follows–what is the time span between the clear local maximum on the curve of daily Conf increases and the consequent local maximum appeared on the curve of daily deaths. This will be indirect evidence of the size of the interval between infection and death.

**Fig. 6:**
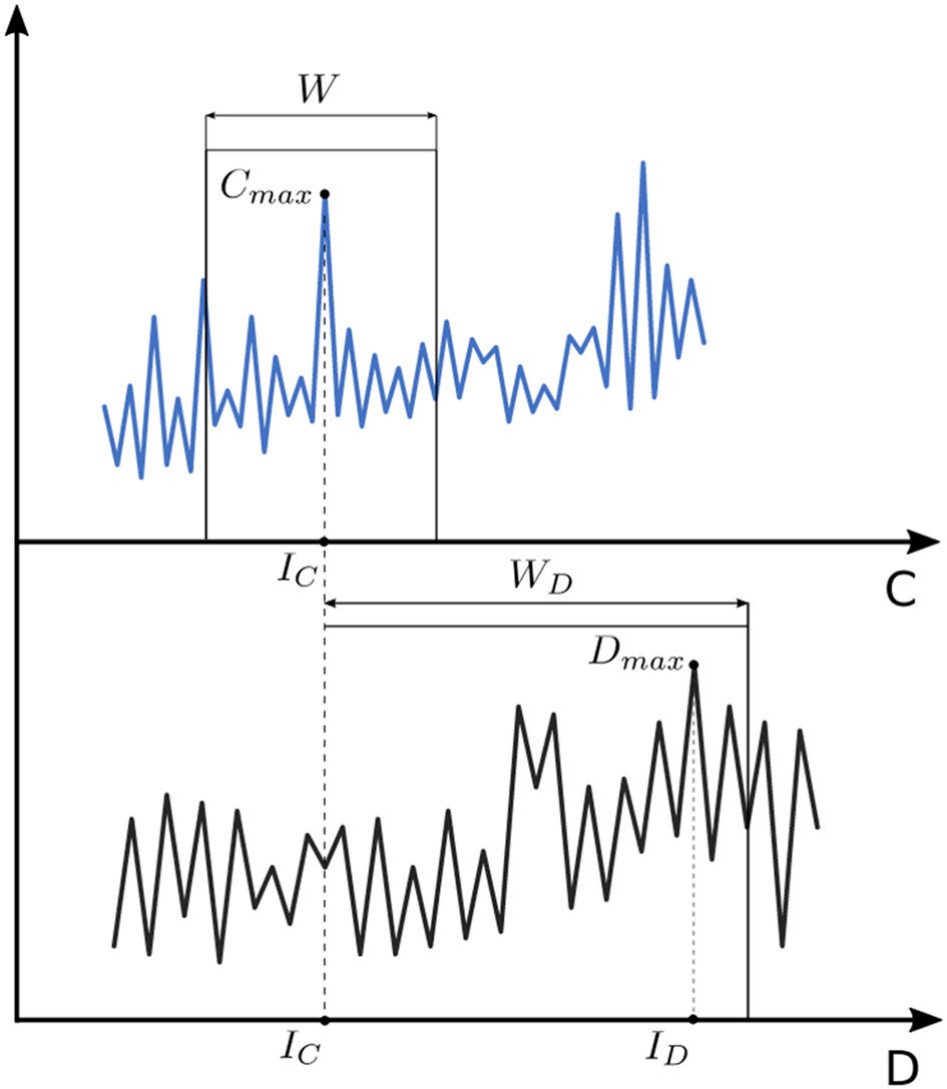
Time windows in the Conf (top) and Dea (bottom) graphs

In its classic application, bootstrapping is used as a method of estimating the value of a certain feature of a set by multiple random sample creation and determining the value of the desired feature for each such implementation (Bollen & Stine 1992; Preacher & Selig 2012, Berkowitz & Kilian 2000). In the case considered here, we will randomly create a window of equal length ${w}_{D}$ for all trials in the Dea series and look for the maximum value in it. The beginning of this window will be specifically defined by the index of the maximum value in a similar window from the length $w$ but in a parallel Conf time series. The relationship between these two windows is explained in Fig. 6.

Figure 6 shows, similarly to Fig. 3, two time series Conf and Dea unshifted with the marked windows $w$ and ${w}_{D}$.

We are looking for the maximum value in each of the windows. First, in the window selected in time series $C$.

3 $$C_{\max } = \max_{i} C_{i}$$

for ${ }i = i_{r} ,i_{r + 1} \ldots { }i_{r} + w$, for which ${ }i_{r} + w < N$.

where $i_{r}$ it is a day number (index in time series) drawn for a given sample.

The index (day number) in the series $C$ for which the maximum value for the window $w$ is found, denote $I_{C}$:

4 $$I_{C} = \arg \max \left\{ {C_{i} :i = i_{r} ,i_{r + 1} \ldots i_{r} + w, i < N} \right\}$$

Starting from the ${I}_{C}$ found in this way, we create another window of length ${w}_{D}$ in the time series $D$ and look for the maximum value in it:

5 $$D_{\max } = \max_{i} D_{i}$$

for $$i = I_{C} ,{ }I_{C} + 1,{ }I_{C} + 2,{ } \ldots { }I_{C} + w_{D}$$ and $$I_{C} + w_{D} < N$$.

Day number in the time series, in which the maximum is obtained, denote $I_{D}$:

6 $$I_{D} = \arg \max \;\left\{ {D_{i} :i = i_{r} ,i_{r + 1} \ldots i_{r} + w, i < N} \right\}$$

In general case, for each sample$s$, $s = 1, 2, ..., S$ obtained in this way—the pair of ${I}_{C}$ and ${I}_{D}$ values will be different. Obviously, the difference of these values will also be different, assuming that we will take into account only those differences for which ${I}_{D}$ occurs after${I}_{C}$, i.e. the day number in the time series is smaller for $I_{D}$.

7 $${\text{dI}}_{s} = I_{D}^{s} - I_{C}^{s} s = 1, 2, \ldots , S$$

for $$\forall_{s} :I_{D}^{s} > I_{C}^{s}$$.

The difference ${\text{dI}}_{s}$ is the examined feature, the distribution of which we are looking for in the multiple examination of the collected samples. For such definitions, simulations were carried out on the basis of bootstrapping for data concerning Poland. Simulations of the next draw for the beginning of the time window in the Conf series were carried out $S = 1000$ and then $S = 100000$ times without any particular statistical differences in terms of the distribution of the examined feature, i.e. differences between local maximums in Conf and Dea series (with the number of iterations, e.g. in Simar & Wilson 2000 and Steedman et al. 2003).

## Results

A research for the all 4 considered countries has been conducted with the described bootstrapping method.

**Fig. 7:**
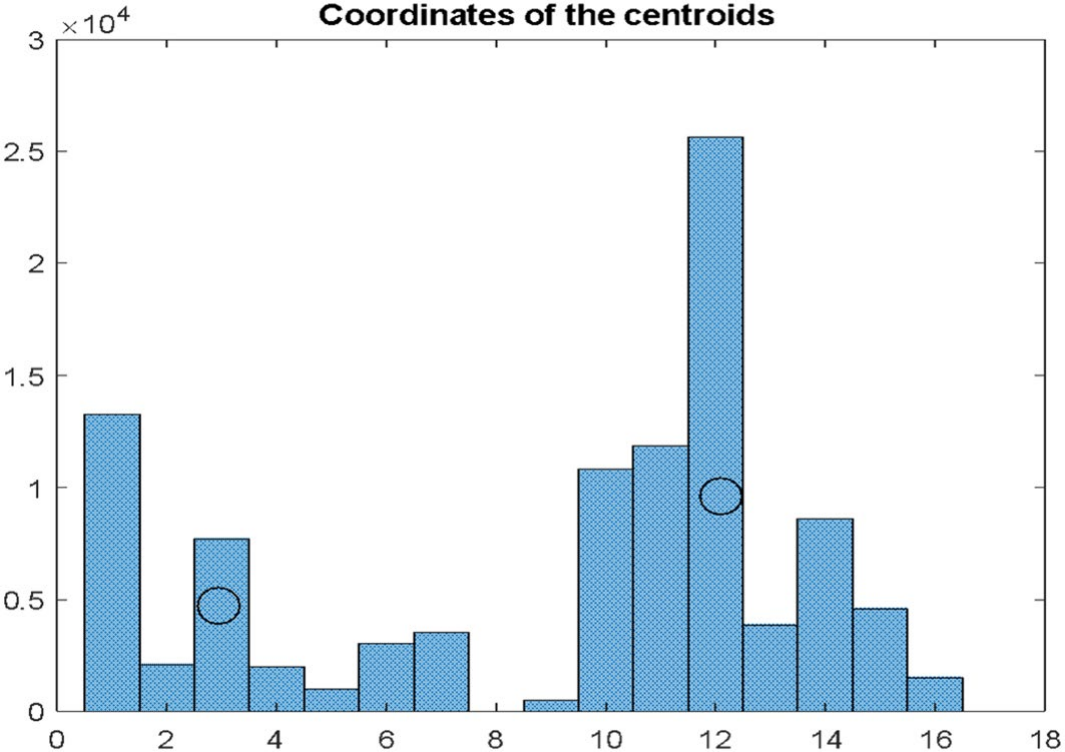
Histogram of differences ${\mathrm{dI}}_{\mathrm{s}}$ for $\mathrm{S }= 1000$ simulations according to the bootstrapping principle in Conf and Dea series for Poland. The histogram intervals were divided into two conventional clusters for which the coordinates of the centroids were determined

The obtained results ${dI}_{s}$ for Poland are presented in the form of a histogram in Fig. 7.

It can be concluded from this graph that determining the delay of the maxima of deaths in relation to the maxima of infections is not easy and unambiguous. For the above histogram, the one-day interval that is a median for the entire distribution occurs on the 12th day after the local maximum for infections. However, if we try to concentrate these local maximums into characteristic clusters, it should be noted that apart from the quite clearly dominant interval at the position of the 12th day after the maximum of infections, there is also a group of maximums a bit earlier—between the 1st and the 7th day. They appear as if clusters, let us denote them ${A}_{1}$ and ${A}_{2}$

$$B_{1} , B_{2} , \ldots B_{7} \subset A_{1}$$

$$B_{10} , B_{11} , \ldots B_{16} \subset A_{2}$$

where $B_{1} , B_{2} , \ldots , B_{16} ,$—appropriate bins of the histogram presented in Fig. 7

Assuming that the distribution of the maximum values on the D curve for the given window will have the form as in Fig. 6 and these will be the two observed clusters ${A}_{1}$ and ${A}_{2}$, we will calculate their coordinates as coordinates of certain conventional centroids:

E_1_ = 2.95 E_2_ = 12.11 H_1_ = 4659 H_2_ = 9554.

According to these coordinates, the position of the centroids is marked on the histogram in Fig. 7. The abscissae of these coordinates indicate the position of the centroids on the time axis, and the ordinates of the intensity (number of occurrences) of the intervals ${dI}_{s}$ in a given cluster. The higher the value of the H_i_ coordinate for a given cluster, the greater the significance of the estimation for the E_i_ coordinate of this cluster.

Perhaps the function of the distribution of the size of the interval between infection and death is multimodal with a greater number of such understood clusters and local maxima. A histogram in Fig. 7 most likely will depend on the size of the examined window, especially the one shifted along the Conf axis, and will depend on the country where such research is carried out. Above assumptions confirm a relationship between ${dI}_{s}$ and the state of the health service. This variable is calculated by bootstrapping technique. Here sampling up to 100,000 times was used. The results do not significantly depend on the number of sample simulations. All these may provide avenues for future research.

Extending the research, let us return to the formula (7), which shows the point of interest for comparison with the maximum on the $C$ axis is the point with the ${I}_{D}$ index—for the maximum value in the window of the series $D$. This particular maximum point can be a coincidence of circumstances—overlapping of several factors causing the highest number of deaths during the given day.

Consider the highest few values in the Dea window, not just the highest one. Mathematically this can be formulated as the result of sorting the Dea ordinates in this window in descending order. For further consideration, we can take several ordinates occupying the highest positions after sorting. The previous approach only considered the first position in such a series—the maximum. In s-simulation it was ${I}_{D}^{s}$ according to (7).

The results of sorting are written as:

8 $$I_{{D_{1} }} = i|D_{i} = D_{\max } \;{\text{for}}\;{\text{all}}\;i\;{\text{from}}\;{\text{the}}\;{\text{range}}\;\left[ {I_{C} ,\;I_{C} + w_{D} } \right]$$

$I_{{D_{2} }} = i|D_{i} = D_{max}$ for all $i$ from the range [ $I_{C}$, $I_{C} + w_{D}$ ] except $i$  = $I_{{D_{1} }}$.

$I_{{D_{3} }} = i|D_{i} = D_{max}$ for all $i$ from the range [ $I_{C}$, $I_{C} + w_{D}$ ] except $i$  = $I_{{D_{1} }}$ or $I_{{D_{2} }}$.

By looking for the distribution of the three highest values in the ${w}_{D}$ window, we can build a histogram with three times higher number of events—also in the bootstrap technique. Let us call this model of studying the distribution of the interval ${dI}_{s}$ as model B. The first one based on the distribution of ${dI}_{s}$ only up to the maximum value in the window ${w}_{D}$ shall be called model A.

**Fig. 8:**
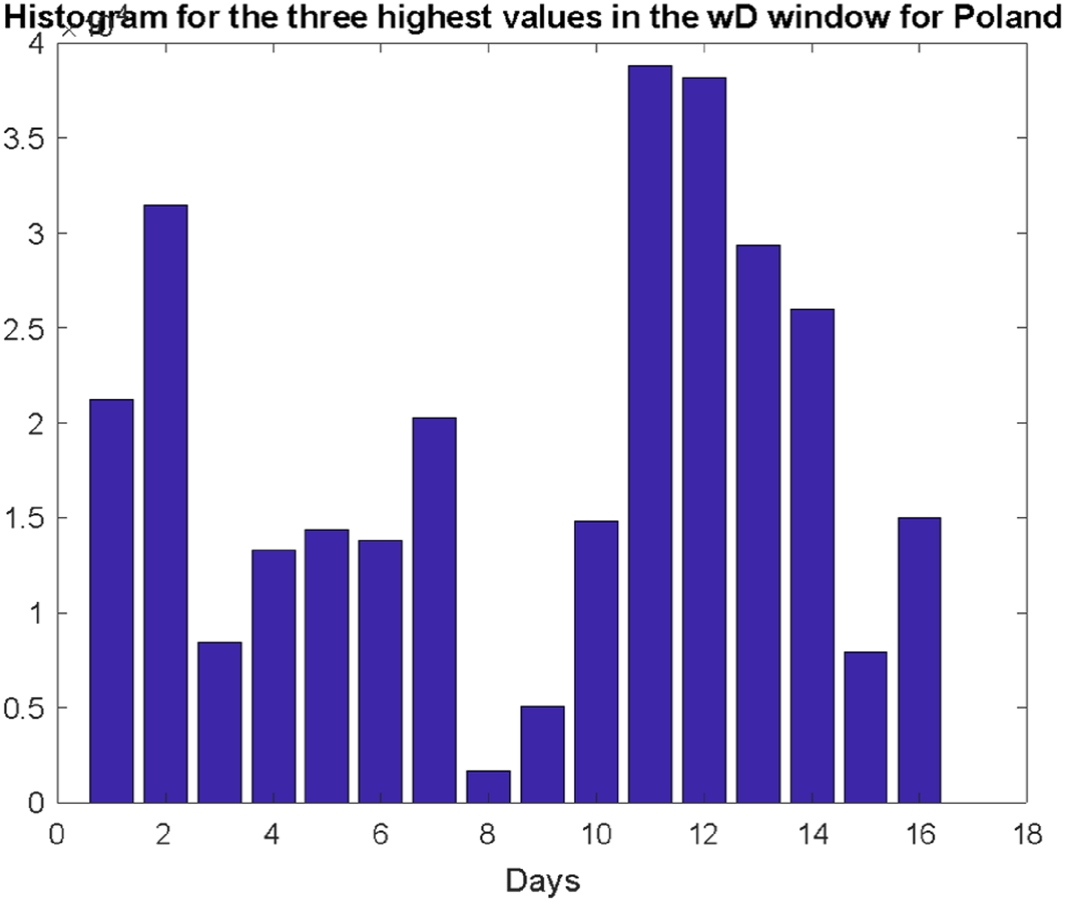
Histogram illustrating the distribution of the three highest values of the number of deaths recorded in the ${\mathrm{w}}_{\mathrm{D}}$ window for Poland. Research carried out according to the model B

In case of Poland, such a histogram, for the same number of simulations $S = 100000$ is presented in Fig. 8. This distribution confirms the hypothesis that the histogram of the interval between infection and death is multimodal.

The most important conclusion of the presented results is that the maximum number of deaths for a country such as Poland will fall on the 12th day (this is the median), but with a fairly significant range of changes as shown in the histogram in Fig. 8. However, generalizing the results of the research, this number will not be clearly dominant, and the distribution of these events (death peaks after local maximum of infections) is rather multimodal.

The authors decided to verify this method also on data from other countries. Several characteristic ones were selected. The USA was selected as a global leader in cases, including over 400,000 deaths (January 2021). India was chosen by its numerous population and relatively weaker health care capacity in comparison to the USA. The third country for which the research was carried out was Germany, the largest European economy and a neighbour of already considered Poland.

**Fig. 9:**
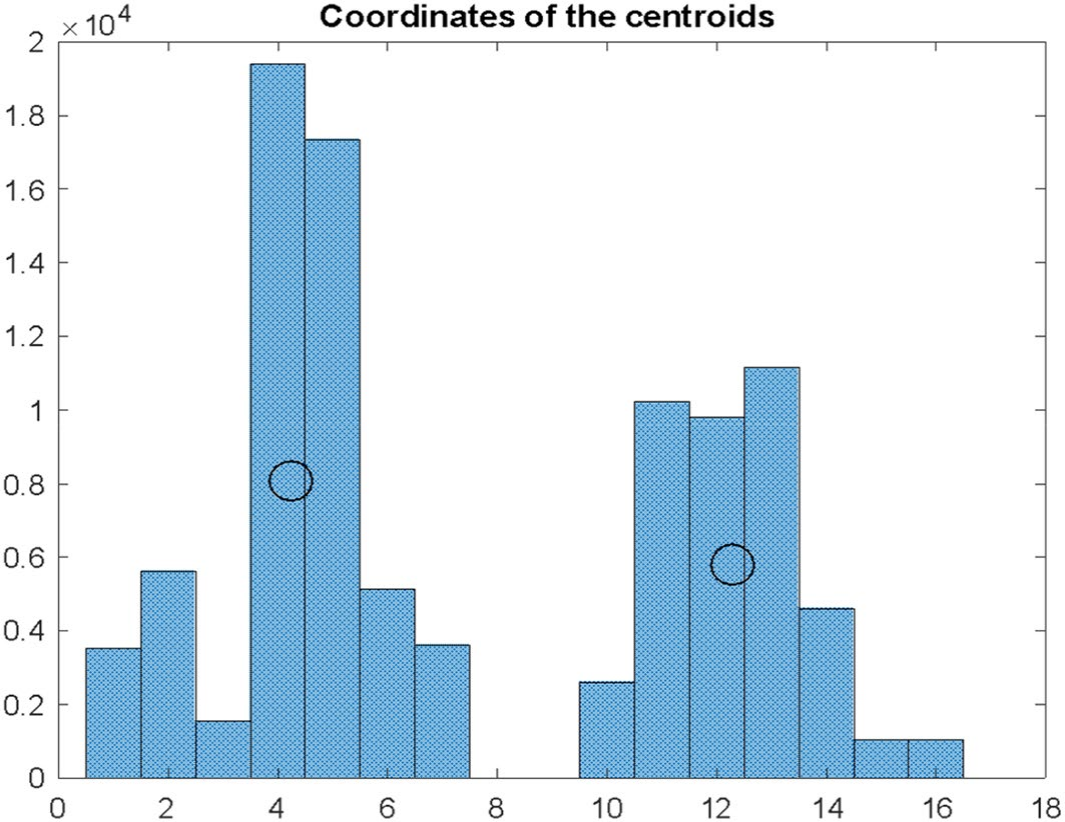
Histogram of differences ${\mathrm{dI}}_{\mathrm{s}}$ for the USA. The histogram intervals were divided into two conventional clusters for which the coordinates of the centroids were determined

The studies conducted for the USA show a similar bimodality (Fig. 9) of the Id interval distribution as for Poland, but with a higher H_1_ height of the first centroid. The coordinates of the centroids were respectively:

E_1_ = 4.26 E_2_ = 12.30 H_1_ = 8044 H_2_ = 5769.

The shift of the centre of gravity of the first cluster with its increased height can be interpreted as a smaller distance between the moment of maximum infection and maximum deaths. Perhaps there should be drawn different conclusions knowing more circumstances and factors on which ${I}_{D}$ depends. The first natural conclusion is that people die faster here Sornette D. and Mearns E. (2020) also agree that the death rate due to COVID-19 in Western countries is, paradoxically, higher due to better health care and a longer life span. A higher average age makes it easier for elderly people to be infected, as they have lower immunity to the coronavirus.

**Fig. 10:**
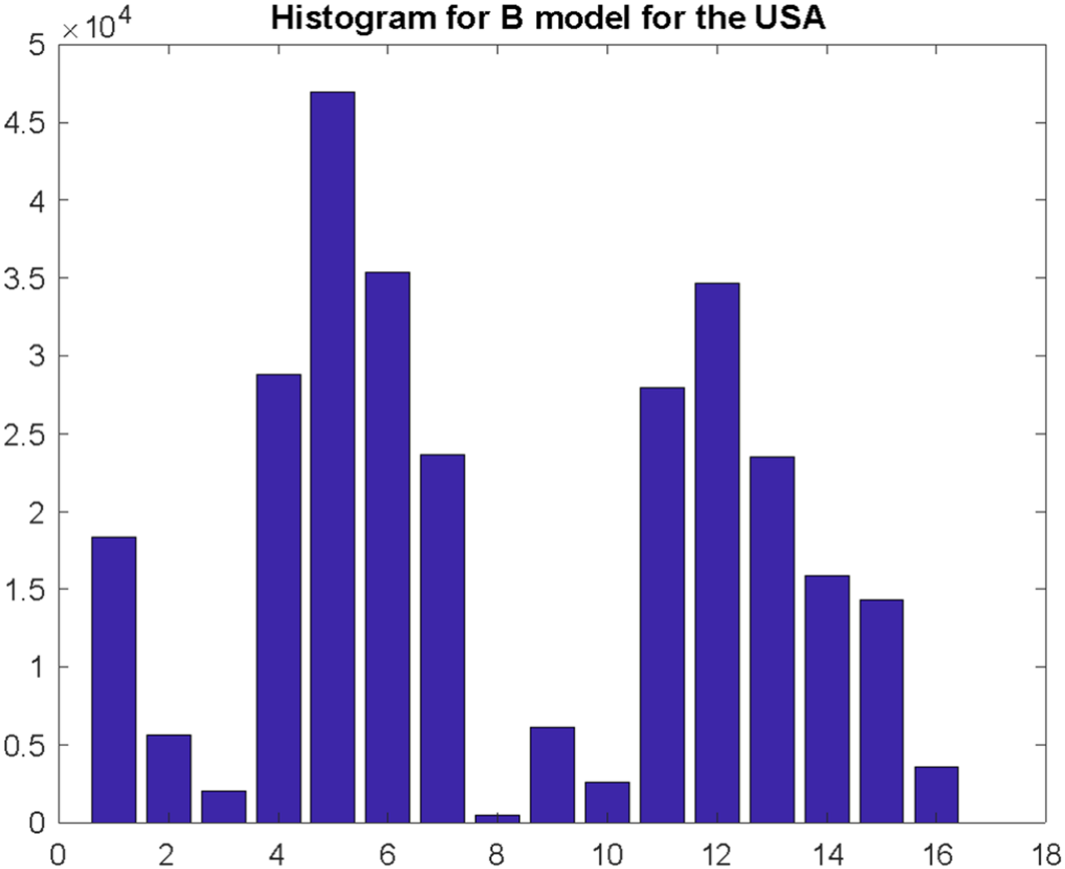
Histogram illustrating the distribution of the three highest values of the number of deaths recorded in the ${\mathrm{w}}_{\mathrm{D}}$ window for the USA. Research carried out according to the model B

Similar to Poland, there were studies for USA carried out according to model B (with three highest maxima), obtaining a repetition of the bimodal distribution (Fig. 10).

**Fig. 11:**
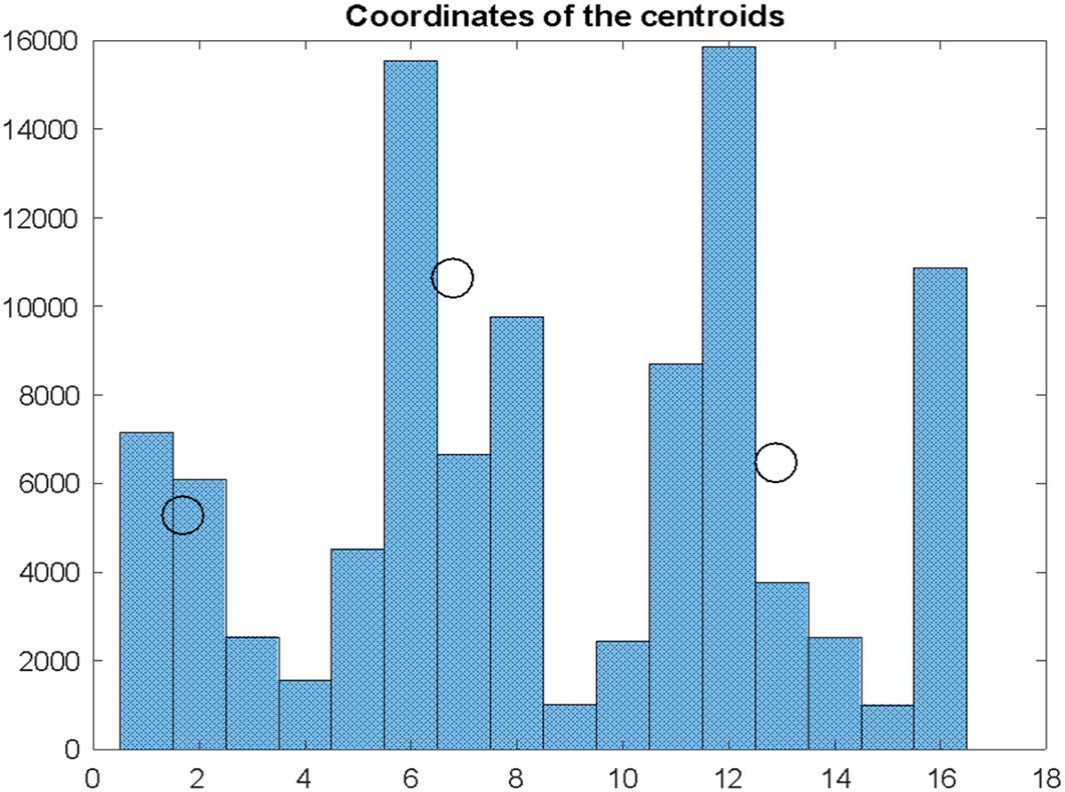
Histogram of differences ${\mathrm{dI}}_{\mathrm{s}}$ for Germany. The histogram intervals were divided into three conventional clusters for which the coordinates of the centroids were determined

**Fig. 12:**
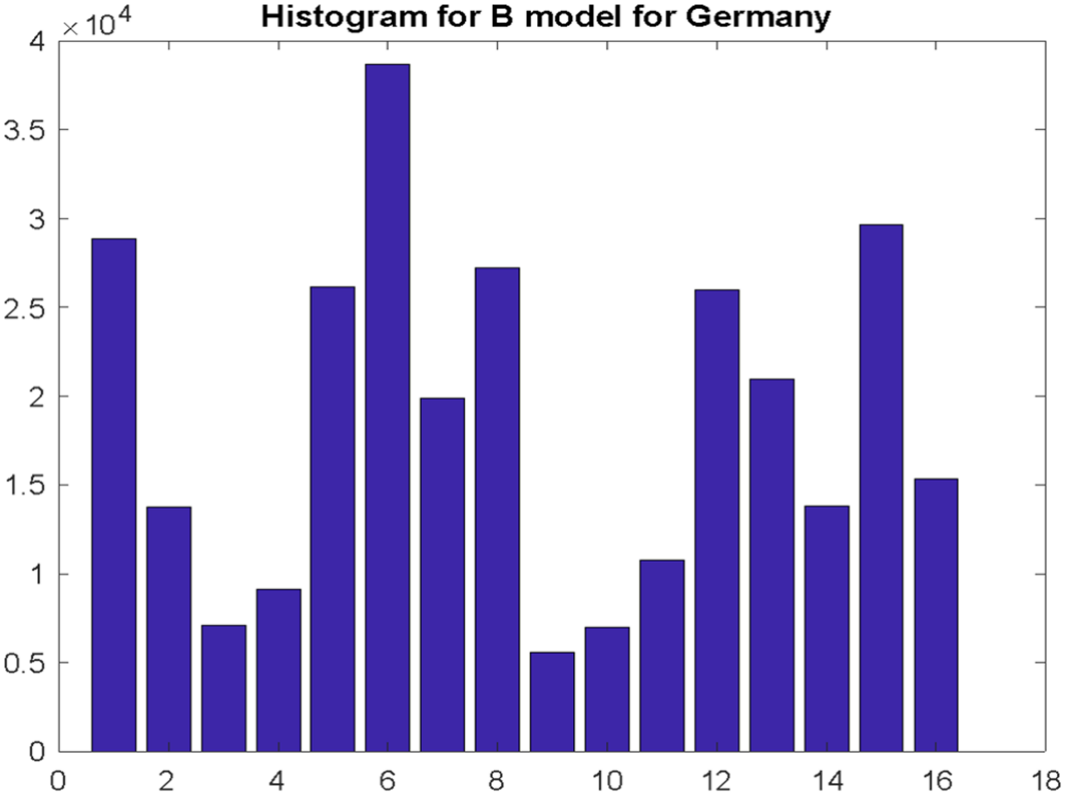
Histogram illustrating the distribution of the three highest values of the number of deaths recorded in the ${\mathrm{w}}_{\mathrm{D}}$ window for Germany. Research carried out according to the model B

The next research has been conducted for Germany. The results are depicted in Fig. 11 and for model B on Fig. 12. A dilemma has been appeared here, whether ${I}_{D}$ distribution for Germany is bi- or trimodal. The authors decided to highlight tree clusters in order to consider such a possibility. However, there has been such a possibility on the model B histogram for the USA, in case of model B for Germany it is clearer.

In the research carried out according to model B, the histogram presented in Fig. 12 was obtained.

E_1_ = 1.71 E_2_ = 6.90 E_3_ = 12.91 H_1_ = 5261 H_2_ = 10612 H_3_ = 6454.

**Fig. 13:**
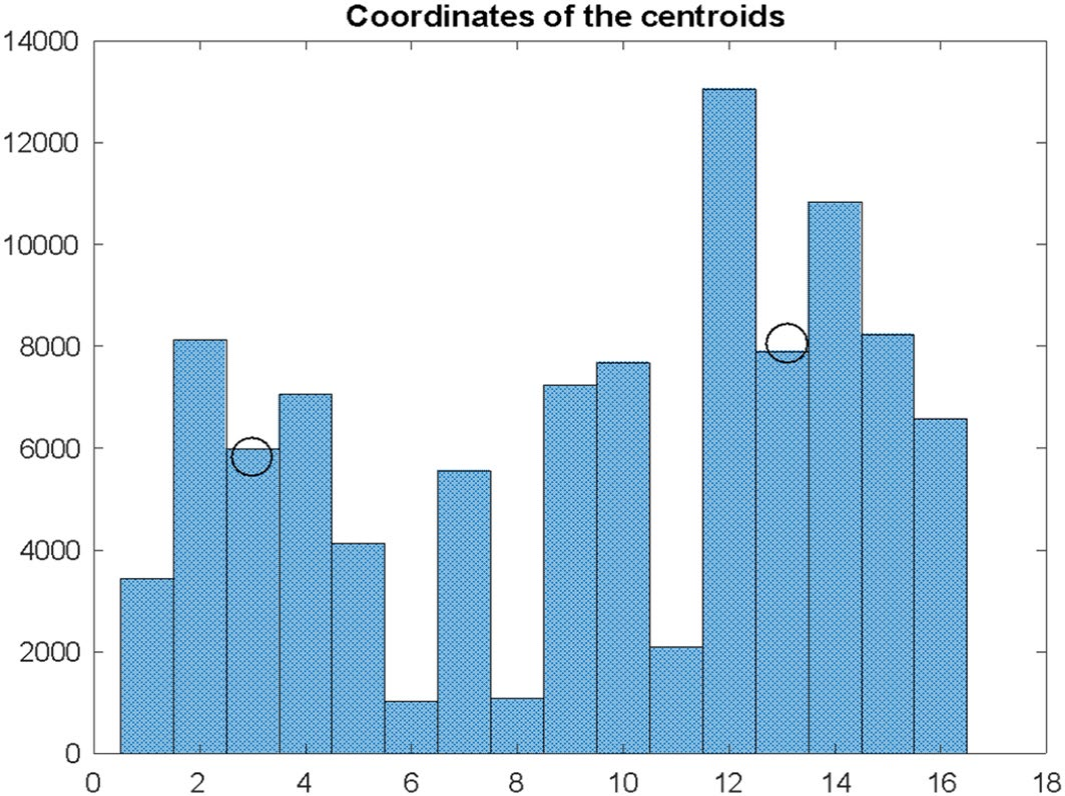
Histogram of differences ${\mathrm{dI}}_{\mathrm{s}}$ for India. The histogram’s intervals were divided into two conventional clusters for which the coordinates of the centroids were determined

**Fig. 14:**
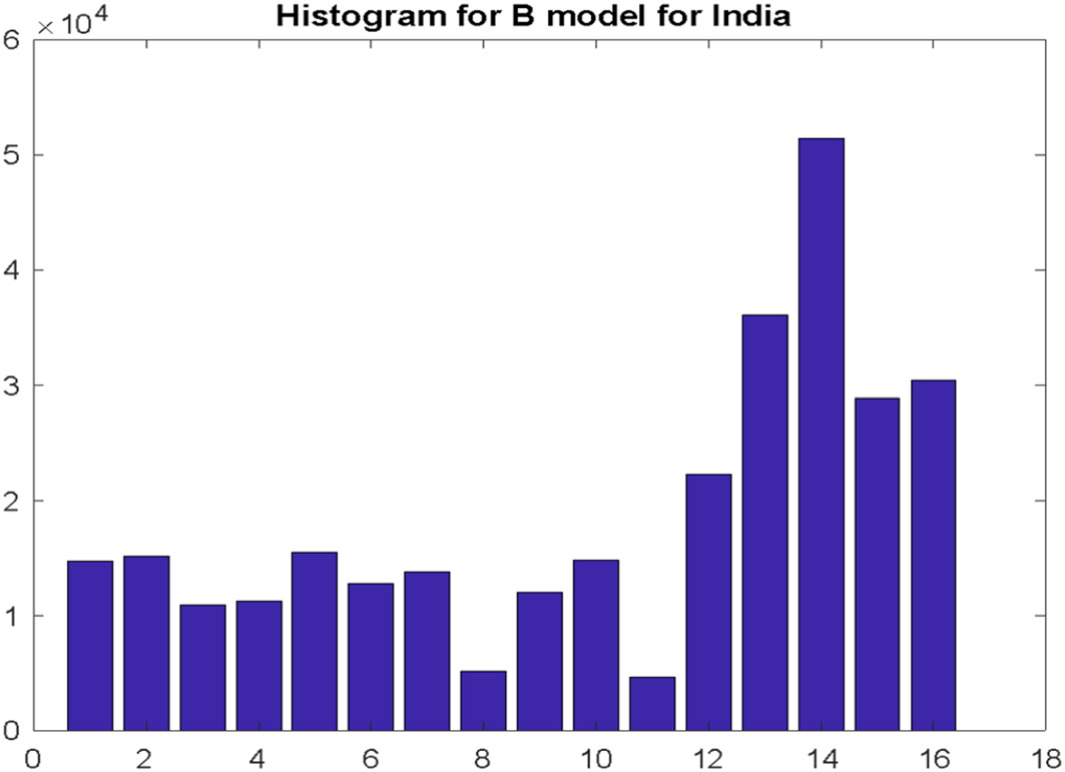
Histogram illustrating the distribution of the three highest values of the number of deaths recorded in the ${\mathrm{w}}_{\mathrm{D}}$ window for India. Research carried out according to the model B

Finally, results for India are presented. There are centroids charts and its coordinates for model A depicted in Fig. 13 and research results for model B on Fig. 14.

A clearly bimodal distribution was obtained in model A and previously unobserved flattening in the initial range of model B.

E_1_ = 3.00 E_2_ = 13.11 H_1_ = 5812 H_2_ = 8032.

Thus, the study of the distribution of time intervals between infections and deaths for the four selected countries was completed.

## Discussion

We have presented a study of time interval between infections and deaths for 4 countries. Selecting Poland is a natural choice due to the authors' origin. Germany is the neighbour country to Poland, but with better healthcare, economy, quality of life and higher personal incomes. Differences are significant and can differ both countries in the studied aspects. The remaining countries were selected due to their global importance. Both USA and India differ in multiple obvious aspects.

Such a large dispersion of the characteristics of these countries indicates the universality of the method. It can be assumed that the method will solve the same problem equally well for any country. Multimodality is probably the result of the overlapping of infections and deaths of different social groups with different statistical and demographic characteristics, such as age, wealth and health. It would be interesting to study the interval between infection and death for a completely homogeneous group, but it would be difficult to find such a selected group in publications.

Perhaps different results would also be obtained for a society with different dynamics of employment during the week, without the typical weekend.

The presence of this particular break in the week in the performance of registration obligations (infections and deaths, or only deaths) can be explained by the presence of zero bins in Figs. 7 and 9 or almost zero bins in Figs. 11 and 13. Let us imagine a clearly dominant value in a series of infections and the repeatability of this dominance on a selected day of the week. For example, in Poland, the days of the week with high rates of infections are the middle days of the week—Wednesday and Thursday. Then, regularly 3–4 days later, the weekend with low deaths (more precisely death registration) falls. In the histogram, in which we will record the events of the maximum deaths, these two days after the maximum infections, e.g. on Thursday, will be rather deprived of a chance that the maximum deaths would occur there.

Considering the influence of various factors on the studied time difference between the statistical infection and death, one should mention the restrictions introduced by governments and local administrations. This relationship has not been investigated here, but a hypothesis of probably high dependence can be put forward. More broadly, it can be assumed that the impact of administrative management, including in particular the management of public health infrastructure, will be extremely important. This task is extremely difficult to optimize, even if there was a consensus as to the infrastructure quality criterion used—for example, minimizing the number of deaths. Neither theoreticians nor management practitioners have solved this task yet.

## Conclusions

The study aims to find a statistical relationship between the moment of infection and the time that elapses before possible death. The correlation-based search method failed due to the dominant influence of the ubiquitous weekly cycle. In this cycle, the results of registration of infections and deaths during the weekend are artificially lowered, basically all over the world. The rejection of the correlation method is an additional result of the performed calculations.

Two models A and B were chosen by abandoning the correlation method.

In the model A, a random time window was created in the series of infections. Starting from the local maxima of infections value within this window, the time window in the series of deaths was created. In the time series of deaths in the second window, we searched for a day with the maxima of deaths. A time interval between maxima in infections and deaths time series was the searched time interval between both events.

In the model B, for the time window created in a time series of infections, like in the model A, a different algorithm of searching for maxima in the time window of deaths was used. Instead of the date of one maxima occurrence, the researchers looked for the three days with the greatest number of deaths. Histograms of ${I}_{D}$ intervals were added together.

Based on the results of the presented simulations the first conclusion that can be drawn is the multimodality of the ${I}_{D}$ distribution. The interval between the times of the local maxima in infections and the potentially associated death peaks is not clear-cut. It seems that such a distribution is the sum of many local distributions in regions and professions (depending on the restrictions applied in a given country).

The approach allowed to obtain independently of time both a result of bootstrapping randomization at many points in time and generalization of the relationship between infections and deaths. The beginning of the time window (within which the relationship between the time of infection and the time of death) was searched for thousands of times.

In all four cases of the countries considered here, the interval ${I}_{D}$= 12 days is repeated fairly precisely as the one with the visible frequency of occurrences.

Without going into a detailed comparative analysis of the countries considered here, one can find rather unexpected distributions. The United States and Germany—well developed countries—have the dominant centroid coordinates located closer to the beginning of the maximum infection countdown reference. The results are presented in Table 1.

**Table 1:** Coordinates of centroids for individual countries

	E1	E2	E3	H1	H2	H3
Poland	2.95	12.11	–	4659	9554	–
USA	4.26	12.30	–	8044	5769	–
Germany	1.71	6.90	12.91	5261	10,612	6454
India	3.00	13.11	–	5812	8032	–

The explanation of these results and their inconsistency with expectations probably requires non-statistical research, which the authors encourage.

Perhaps the justification of the differences should be searched in a way suggested in referenced works of Chiara Sotis i Salvatore F. Pileggi et al. (2021) and also in work of Sornette D. and Mearns E. (2020), that is about the analysis of the factors influencing infections and deaths in geographical blocks, adequately presented in the table above.

## Data Availability

Data available from the public sources.
